# Preclinical *in vivo* Performance of Novel Biodegradable, Electrospun Poly(lactic acid) and Poly(lactic-co-glycolic acid) Nanocomposites: A Review

**DOI:** 10.3390/ma8084912

**Published:** 2015-08-03

**Authors:** Claudia Holderegger, Patrick R. Schmidlin, Franz E. Weber, Dirk Mohn

**Affiliations:** 1 Private Practice, Marktgasse 27, 8400 Winterthur, Switzerland; E-Mail: c.holderegger@bluewin.ch; 2 Clinic of Preventive Dentistry, Periodontology and Cariology, Center for Dental Medicine, University of Zurich, Plattenstrasse 11, 8032 Zurich, Switzerland; E-Mail: dirk.mohn@chem.ethz.ch; 3 Department of Cranio-Maxillofacial and Oral Surgery, Oral Biotechnology and Bioengineering, Center for Dental Medicine, University of Zurich, Plattenstrasse 11, 8032 Zurich, Switzerland; E-Mail: franz.weber@zzm.uzh.ch; 4 Institute for Chemical and Bioengineering, Department of Chemistry and Applied Biosciences, ETH Zurich, Vladimir-Prelog-Weg 1, 8093 Zurich, Switzerland

**Keywords:** 3D scaffold, biodegradable polymer, bone, calcium phosphate, calvarial defect, electrospinning, experimental animal models, nanocomposite

## Abstract

Bone substitute materials have witnessed tremendous development over the past decades and autogenous bone may still be considered the gold standard for many clinicians and clinical approaches in order to rebuild and restore bone defects. However, a plethora of novel xenogenic and synthetic bone substitute materials have been introduced in recent years in the field of bone regeneration. As the development of bone is actually a calcification process within a collagen fiber arrangement, the use of scaffolds in the formation of fibers may offer some advantages, along with additional handling characteristics. This review focuses on material characteristics and degradation behavior of electrospun biodegradable polyester scaffolds. Furthermore, we concentrated on the preclinical *in vivo* performance with regard to bone regeneration in preclinical studies. The major findings are as follows: Scaffold composition and architecture determine its biological behavior and degradation characteristics; The incorporation of inorganic substances and/or organic substances within composite scaffolds enhances new bone formation; L-poly(lactic acid) and poly(lactic-co-glycolic acid) composite scaffolds, especially when combined with basic substances like hydroxyapatite, tricalcium phosphate or demineralized bone powder, seem not to induce inflammatory tissue reactions *in vivo*.

## 1. Introduction

The reconstruction of bone that has been lost due to pathologic changes or injury is a major interest in preclinical and clinical research and has led to the development of a plethora of materials that should help to efficiently regenerate or at least repair bone defects [1,2,3]. Autografts still remain the gold standard material, *i.e.*, the harvesting of bone from the patient but from a non-injured site, but the need for a second intervention procedure, donor site morbidity, and an often limited supply of bone are associated with this intervention and limit this approach [1]. Alternatives are therefore allografts, *i.e.*, bone material from an individual of the same species, but again this may entail other problems such as rejection or disease transmission [4].

As an alternative to these tissue-based strategies, synthetic bone substitutes that act as scaffolds can be used and implanted. Whereas metals are the material of choice for load-bearing indications, due to their good mechanical properties, ceramics exhibit a higher biocompatibility as their chemical composition resembles the mineral phase of bone tissue. However, both materials are generally poorly degradable and, thus, not used for smaller defects, and they cannot be used as solid body implant materials.

Biodegradable polymers, such as poly(lactic acid) (PLA) and the co-polymer poly(lactic-co-glycolic acid) (PLGA) have been widely investigated and applied to fabricate porous scaffolds in order to restore damaged tissue [5,6]. A variety of biomedical materials have been developed to fulfill the mechanical and biological demands that the various tissues require. Amongst these, a flexible, moldable, electrospun cotton wool-like nanocomposite has been proposed [7,8,9]; it incorporates amorphous calcium phosphate nanoparticles into a biodegradable synthetic PLGA. This material is prepared through an electrospinning process, which gives it the typical cotton wool-like appearance. This characteristic of the material allows easy proportioning, handling, and adaption to any bone defect. Preclinical studies have shown high bioactivity of this material as soon as four weeks after implantation, with the formation of new bone and increased cell density. Resorption of the graft material as early as four weeks after surgical placement was also reported [9]. The authors highlighted the need for further investigations in animal models to evaluate the long-term stability and clinical outcomes of this material. Since then, many more study groups have assessed similar scaffolds based on PLA and PLGA as a carrier material in an electrospun form that can be used as a carrier for many inorganic materials in an appropriate size and form, mostly incorporated as nanoparticles.

As PLA and PLGA degrade mainly by hydrolysis, there is still some concern regarding the host tissue response to their degradation products. These metabolites have been shown to exhibit a toxic influence on cell culture systems *in vitro* for high concentrations [10]. Despite the fact that there is a large number of materials on the market, the present review aims to summarize, in the first part, the most important aspects of the applied biodegradable materials and, in the second part, to screen the literature with regard to the biocompatibility of these materials when implanted in animals, given the still remarkably controversial background on the degradation of PLA- and PLGA-based electrospun materials.

## 2. Material Characteristics and Degradation Behavior

Nowadays, medical implants are used to help prolong the lifespan of humans and facilitate the life of elder people. Biomaterial science is characterized by the search for improved biocompatibility, enhanced cell-material interaction, tailored degradation, integrative biomaterials design, and other specific properties [11] of polymers, metals, and ceramics. These three materials represent the classes that are used to create biomaterials either as a pure material or, most of the time, as a composite product.

Polymers represent a mere organic matrix and the physical and chemical properties vary tremendously, depending on the application area, like wound dressing, orthopedics, cardiovascular interventions, or drug delivery. This material class can be subdivided into two categories that are important for medical devices, biodegradable *vs.* non-biodegradable polymers. One of the main advantages of biodegradable polymers is the prevention of implant removal and the circumvention of a persistent foreign body; plus, these polymers can be engineered so that they degrade at a certain rate in order to transfer load to a healing bone [12,13]. Biodegradable polymers are further divided into naturally derived materials, including proteins or polysaccharides, and synthetically prepared materials, mainly aliphatic polyesters. Focusing on the latter polymer, some of the most often-used materials are saturated poly-α-hydroxy esters, including poly(glycolic acid) (PGA), PLA, and the co-polymer thereof PLGA [6]. The clearage of the degradation products by natural pathways out of the body and the long history of use might be the reasons that they are the most commonly used and the most widely investigated degradable polymers. PGA is a highly crystalline aliphatic polyester with a high melting point and low solubility in organic solvents. It is more hydrophilic than PLA and degrades faster. PLA appears mainly as L-PLA (PLLA), D-PLA (PDLA), and as racemic mixture D,L-PLA (PDLLA). The optically inactive form, PDLLA, is always amorphous, while the two others are semicrystalline [14]. This difference also determines the application areas for the various PLA forms. PDLLA is often considered as a drug delivery vehicle, due to its monophasic form, while PLLA is preferred for applications where a high mechanical strength is necessary. It is possible to adapt the degradation rate for a specific application by the combination of PLA and PGA. It has to be noted that a 50/50 copolymer ratio of lactic and glycolic acid has the fastest degradation rate, yet there is no linear relationship for the degradation kinetics of the two components [15]. In general, the physical and mechanical properties are adjustable and depend on the molecular weight, the polydispersity, and the co-polymer ratio. However, the degradation rates depend not only on the molecular weight but also on the environmental conditions, the device size and form, and on any additives in the polymer.

Most of the polymers, although partially hydrophobic, have a hydrophilic nature, which is dominant enough that the rate of water penetration into the material excels the rate of degradation. Therefore, they undergo mostly a bulk erosion process. However, the characteristic size of a device can also lead to a surface erosion process [16]. This fact has to be taken into account when designing a device for a specific application. Although there are different mechanisms of erosion [14], the main cause for the aliphatic polyesters described here is the hydrolytic degradation by de-esterification of the polymer backbones. As the degradation proceeds, the carboxylic end groups auto-catalyze the degradation process via the low pH and the cleavage of the backbone is enhanced [17].

Of particular interest for the design of a medical implant, next to the degradation rate (tailored by additives or the polymer chain length), is the size of the polymer matrix (morphology) itself [18] and, of course, the polymer composition [19]. The acidic degradation products of PLA, PGA, or PLGA can, on the one hand, induce an early failure of the implant, and can, on the other hand, start an adverse tissue reaction in the body [20], but do not necessarily have to [21]. Athanasiou *et al.*, reported different results (with and without inflammation) using PLLA at different sites in rat models [22]. Often other factors, such as leachable impurities from the synthesis, can also trigger inflammation [23]. Polymer additives are often of an inorganic nature, namely a ceramic such as tricalcium phosphate (TCP), hydroxyapatite (HA), or bioactive glass (BG), and are either used to dose the degradation [24] and/or to counteract the effects of the degraded by-products due to their alkaline nature. Additionally, polymers can benefit from these additives as they render the composite material so-called bioactive, which is the ability of the composite material to bond to bone tissue [25]. This term is generally used within biomineralization studies and describes the ability of a material to form calcium phosphate depositions when immersed in simulated body fluids.

**Figure 1:**
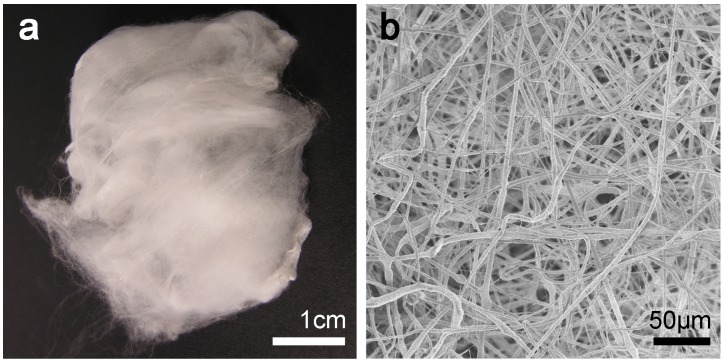
(**a**) Photographic image of a flexible electrospun poly(lactic-co-glycolic acid)/tricalcium phosphate (PLGA/TCP) (60/40) composite material. (**b**) Electron microscopy image of an electrospun PLGA/TCP (60/40) composite material showing an open and porous structure.

Several techniques are applied to combine the organic and inorganic materials in order to better suit the demands of a specific application [6]. Today, various shapes of foams, meshes, films, fibers, or microspheres are manufactured with the ulterior motive to be dedicated to a specific utilization. In order to take advantage of a high surface to volume ratio and, thus, of a possible higher reactivity potential, fibers offer a tremendous advantage over films or rigid blocks. Additionally, the manufactured composite is highly flexible and shapeable. This advantage can be of importance for bone tissue engineering as the operation site is sometimes difficult to reach. The method of choice today to produce ultrathin fibers is electrospinning [26]. This tool allows the preparation of open-structured and highly flexible scaffolds for applications in filtration, wound dressing, tissue engineering, or reinforcement. Furthermore, a fibrous architecture can positively affect cell ingrowth [26]. Electrospinning of various polymer systems and composites into mats, meshes, and scaffolds for tissue engineering and drug delivery has been described in detail [27,28]. Further, it has been shown that incorporating inorganic particles, preferentially in a nanoparticulate form to achieve a homogenous distribution (Figure 1), with biodegradable polymers enables the production of highly flexible and reactive nanocomposites [7,29] that can be advantageous for tissue engineering applications. Several *in vitro* studies have shown that electrospun composite materials performed well, but only a few *in vivo* studies have been conducted so far. Whether the acidic degradation products of the biodegradable PLA or PLGA can trigger inflammatory reactions and whether electrospun materials can perform well are still on-going research topics in biomaterials science.

## 3. Biocompatibility in Preclinical Studies

### 3.1. Aim of the Review on Biocompatibility

This review was performed in order to specifically study the biocompatibility of electrospun PLA and PLGA scaffolds for bone regeneration when applied *in vivo*, focusing on preclinical studies. In addition, the potential effects of scaffold-related factors (e.g., molecular weight, co-polymer composition, and fiber diameter of PLA and PLGA), host-related factors (e.g., animal model or defect type), and possible tissue reactions on the degradation process of PLA and PLGA were also evaluated if applicable.

### 3.2. Search Strategy

An electronic search of the published literature was conducted on the following databases: Embase, Medline, PubMed Premedline, Biosis Previews, and Scopus. Papers were included if published by April 2015.

The following key words, separately or in combination, were used: (((glycolic[tiab] OR polyglycolic[tiab]) AND (lactic[tiab] OR polylactic[tiab])) OR (lactic acid[tiab] AND poly[tiab]) OR polylactate[tiab]) AND (electrospun[tiab] OR electrospinning[tiab] OR fibrous[tiab] OR nanofibrous[tiab] OR fiber[tiab] OR fibers[tiab] nanofiber[tiab] OR nanofibers[tiab]) AND (bone[All Fields] OR osseous[All Fields]) AND (mouse[All Fields] OR mice[All Fields] OR rat[All Fields]OR rats[All Fields] OR hamster[All Fields] OR hamsters[All Fields] OR guinea pig[All Fields] OR guinea pigs[All Fields] OR monkey[All Fields] OR monkeys[All Fields] OR rabbit[All Fields]OR rabbits[All Fields] OR human[All Fields] OR humans[All Fields] OR animal[All Fields] OR animals[All Fields]) AND (publisher[sb] OR inprocess[sb]).

### 3.3. Review Process

Two independent reviewers (Claudia Holderegger and Patrick R. Schmidlin) performed the assessment of eligibility and data extraction. Any disagreement was resolved by discussion and, if necessary, by communication with a third reviewer (Dirk Mohn).

Initial screening of titles was followed by an abstract screening using the following inclusion criteria: publication in German or English language; animal clinical trials; electrospun PLA or PLGA scaffolds when explicitly used for guided bone regeneration in critical or non-critical bone defects; assessment of the amount of new regenerated bone and the registration of possible tissue reactions. Studies were excluded for the following reasons: human studies, case reports, reviews, bone regeneration with non electrospun PLA or PLGA, or electrospun PLA or PLGA scaffolds mixed or coated with organic substances without pure PLA or PLGA control groups.

Subsequently, full text of all possibly relevant papers were checked for the fabrication and the characterization of the PLA and PLGA scaffolds, the *in vivo* experimental model, the defect type, the methods of evaluating bone regeneration, the methods of evaluating inflammation reactions and histological assessments, and the obtained results. Duplicate articles were identified and removed.

### 3.4. Results

During the initial search, 839 references were identified. After the screening of these titles, abstracts and full texts, 10 papers could be found [9,30,31,32,33,34,35,36,37,38], which finally formed the basis of this systematic review.

#### 3.4.1. Description of Materials

Within the identified literature, seven studies examined PLLA [30,31,32,34,35,37,38] whereas only three studies used PLGA [9,33,36].

Two studies reported a ratio of 85:15 regarding their components of PLGA, which relates to a co-polymer composed of 85% lactic and 15% glycolic acid [9,36]. One study did not declare the exact composition of the used PLGA [33]. All lactic acid polymers consisted of L-lactic acid, PLLA [30,31,32,34,35,37,38].

One factor influencing the degradation process of the PLLA/PLGA-containing scaffolds is the molecular weight, which is usually given in Da or g/mol. By increasing the molecular weight of conventional PLGA from 10–20 kDa to 100 kDa, degradation rates can change from several weeks to several months [39,40]. One study used PLGA of 80,000 Da [36], whereas another study used PLGA with different molecular weights (380,300 g/mol to 181,900 g/mol) [9]. One investigation used PLLA of 300,000 Da [35]. All other studies did not report about the molecular weight of the applied materials.

Some manufacturers characterized their product declaring its viscosity, which is influenced by various factors such as temperature, polymer concentration, polymer chain length, and applied solvent, yet it is correlated to the molecular weight. The viscosity of PLLA solutions significantly varied in this review from 0.9 to 8.2 dL/g [31,32,37,38]. According to the manufacturer’s datasheet the 0.9 dL/g correlates to about 150,000 Da.

In addition, as the scaffold architecture affects PLLA/PLGA degradation and the biological behavior depends also on the accessibility of water, blood vessels, and cells, the fiber diameter plays an important role. The studies in this review reported about fiber diameters of PLGA ranging from 300 nm [33,36] to 10 μm [9], whereas PLLA fibers varied from 300 nm [30,31,34,37] to 7 μm [31,32,38].

With regard to tissue engineering, the porosity of scaffolds strongly influences the diffusion area and, thus, the flow rate of nutrients and metabolic products throughout the scaffolds [41]. As a consequence of this, porosity facilitates the process of local vascularization that is essential for tissue growth and, *vice versa*, porosity can affect the mechanical strength of scaffolds. The scaffold architectures of the studies in this review are described in Table 1. None of the studies declared the pore diameter of the electrospun fibers. However, the orientation of fibers in the scaffold profoundly affects cell migration as well. Lee *et al.* [38] showed that human mesenchymal stem cells migrated 10.46-fold faster along the parallel direction than along the perpendicular direction on PLLA nanofibers. Electrospun scaffold parameters, such as the solution concentration, the solvent properties, the voltage, the solution flow rate, and the distance between needle type and collector, influence fiber characteristics and orientation and, as a consequence of this, the scaffold architecture [42].

To improve mechanical and biological properties of PLLA/PLGA scaffolds, inorganic additives are often used. They can either be used as reinforcing material of the scaffold or as coating material to improve bone tissue response towards PLLA/PLGA scaffolds. In this review, PLGA scaffolds were used with willmite [33], HA [36], or TCP [9]. As an organic adjunction, one PLGA study used simvastatin (SIM) [36].

PLLA scaffolds were combined with HA [32,34,35], TCP [34], or BG [34]. Organic materials like dopamine (DA) [38], demineralized bone powders (DBP) [37], and bone morphogenic protein-2 (BMP-2) [30] were additionally used to improve cell interaction on the surface of PLLA biomaterials.

In this regard it is important to know the relevant information on the composition and architecture of scaffolds because this highly influences the biological behavior. Unfortunately, important information about polymer construction or molecular weight was often missing, complicating the overall scientific comparison of the materials in more detail.

#### 3.4.2. Description of Experimental Methods

All pre-clinical *in vivo* experiments in this review used a calvarial defect model (Table 2). All of them were performed on rats [30,32,33,34,35,36,37], rabbits [9,31], or mice [38]. Six of them used a calvarial critical size model [30,33,34,35,37,38], which means that bone defects were too large-dimensioned for spontaneous bone healing. Non-critical size defect models were chosen in the remaining four studies [9,31,32,36]. The time of evaluation or sacrificing the animals was four [9,30,31,32,36], six [35], eight [30,33,34,36,37,38], 10 [35], or 12 weeks [30], respectively. All defects were sutured and resulted in closed defect healing. Adegani *et al.* [33] were the only authors who did not declare the procedure of wound closure in their study explicitly. One experiment [37] covered the implanted defects with a polyvinyl membrane to minimize the effect of self-renewal capability by the pericranium. Evaluation methods of newly formed bone and the biological behavior of the PLLA/PLGA scaffolds comprised multi-slice spiral-computed tomography [33,34], micro-computed tomography (micro-CT) [9,35,36,37,38] digital mammography [34], radiographic analysis [9,35], scanning electron microscopy [38], hematology, or biochemistry [35]. All studies performed a histological evaluation [9,30,31,32,33,34,35,36,37,38].

**Table 1:** Description of L-poly(lactic acid)/poly(lactic-co-glycolic acid) (PLLA/PLGA) materials and scaffold characterization.

Author	Scaffold Components	Scaffold Architecture	Fiber Diameter
Adegani *et al.* [33]	PLGA 15% (wt/wt) solution dissolved in DMF/THF coating with willmite nanoparticles	porous structure	300 ± 500 nm; willmite coating did not affect fiber diameter
Dinarvand *et al.* [34]	PLLA dissolved in chloroform with a 4% (w/v) concentration coating with HA, BG, TCP; HA + BG	nanofibrous structure with homogeneous distribution of bioceramics along the surface of PLLA	822 ± 97 nm
Jaiswal *et al.* [35]	PLLA with molecular weight 300,000 Da blend with G (3:1) composited with HA	no information	no information
Jiang *et al.* [36]	PLGA (85:15) 10% with molecular weight of 80,000 Da dissolved in a mixture of chloroform + DMF (1:1) mixed with HA (20:1) mixed with HA + SIM (20:1:1)	scaffolds with smooth and nanofibrous morphology	PLGA: 550 ± 50 nm PLGA + HA: 240 ± 30 nm PLGA + HA + SIM: 270 ± 30 nm
Lee *et al.* [38]	PLLA (5.7–8.2 dL/g viscosity; Resomer L 214 S) dissolved in HFIP (2 wt % for random, 2.5 wt % for aligned fibers) coating with polydopamine	scaffolds with random and aligned fiber orientation	1 μm in both structures
Ko *et al.* [37]	PLLA (3.3–4.3 dL/g viscosity; Resomer L 210 S) dissolved in trifluorethanol mixed with DBP (1.0:0.2)	nanofibrous scaffold with randomly oriented fibers with a homogeneous distribution	300–700 nm
Schneider *et al.* [9]	PLGA (Resomer) (85:15) with a molecular weight of 380,300 g/mol and 181,900 g/mol blend with TCP nanoparticles (40 wt %)	fibers exhibiting a porous structure, TCP-containing fibers revealed an increased roughness	5–10 μm
Schofer *et al.* [30]	PLLA (Resomer) 4% (w/w) dissolved in DCM incorporation of BMP-2	three-dimensional non-woven network of nanofibers, fibers showed a porous structure	775 ± 294 nm
Shim *et al.* [31]	PLLA (intrinsic viscosity 0.63 dL/g, molecular weight: 250,000 g/mol); 8% PLLA dissolved in DCM/HFIP or in DCM/DMF or in DCM/acetone with volume ratios (90:10) 3% PLLA in DCM/HFIP (90:10)	PLLA mixture below 2% w/v resulted in beaded fibers, for concentrations > 4%, the fibers fused at the contact points	400 nm–7 μm
Yanagida *et al.* [32]	PLLA (Lactel: intrinsic viscosity: 0.9–1.2 dL/g) dissolved in DMC at 15 wt % mixed or coated, or mixed and coated with HA nanocrystals	PLLA/HA nanocomposite fibers, where HA nanocrystals were mixed into the PLLA matrix as well as coated onto the PLLA surface had submicron-sized dimples on their surfaces	PLLA fibers: 6.1 ± 1.9 μm PLLA/HA mixed: 7.6 ± 1.9 μm

BG: bioactive glass; BMP-2: bone morphogenetic protein 2; DBP: demineralized bone powder; DMF: dimethylformamide; G: gelatin; HA: hydroxyapatite; HFIP: hexafluoroisopropanol; DCM: dichloromethane; SIM: simvastatin; TCP: tricalcium phosphate; THF: tetrahydrofurane.

**Table 2:** Description of *in vivo* experiments with PLLA/PLGA scaffolds.

Author	Animal Model	Defect Size (Diameter) and Wound Treatment	Time of Evaluation	Methods of Evaluation	Area of Regenerated Bone	Histological Results
Adegani *et al.* [33]	rats	8 mm calvarial critical size defects, precise treatment of the wound is not described	8 weeks	MSCT histology evaluation by two independent radiologists	PLGA + willmite: 70% PLGA: 35% Empty: 5%	No sign of inflammation
Dinarvand *et al.* [34]	rats	8 mm calvarial critical size defects, wound was closed with sutures	8 weeks	MSCT Digital mammo-graphy histology evaluation by two independent radiologists	PLLA-HA-BG: 63% PLLA-TCP: 44% PLLA-HA: 23% PLLA-BG: 20% PLLA: 13% Empty: 12%	No sign of inflammation
Jaiswal *et al.* [35]	rats	5 mm calvarial critical size defects, pericranium and skin was closed in layers	6 and 10 weeks	Micro-CT digital X-ray, hematology and serum biochemistry histology evaluation with an image software	6 weeks: PLLA-G-HA: ≈94% PLLA-HA: ≈64% Empty: 30% PLLA: 26% PLLA-G: 13% 10 weeks: PLLA-G-HA: 98% PLLA-G: 80% PLLA-HA: 76% PLLA: 60% Empty: 34%	No sign of inflammation
Jiang *et al.* [36]	rats	5 mm calvarial defects, wound was closed with sutures	4 and 8 weeks	Micro-CT histology evaluation with an image software	4 weeks: PLGA-HA-SIM: ≈4.2% PLGA-HA: <1% Empty: <1% 8 weeks: PLGA-HA-SIM: ≈10% PLGA-HA: <4% Empty: <2%	-
Lee *et al.* [38]	mice	4 mm calvarial critical size defects, wound was closed with sutures	8 weeks	Micro-CT SEM histology precise method of evaluation is not described	PLLA + DA aligned fibers: 28.86 ± 6.5% PLLA + DA random fibers: 10.58 ± 0.9% PLLA aligned fibers: 5.25 ± 3.7% PLLA random fibers: 3.35 ± 1.8%	No sign of inflammation
Ko *et al.* [37]	rats	8 mm calvarial critical size defects, a polyvinyl membrane was laid over the defects and the wound was closed with sutures	8 and 12 weeks	Micro-CT nhistology precise method of evaluation is not described	8 weeks: PLLA: minimal newly formed bone PLLA + DBP: greater extent of newly formed bone than PLLA alone 12 weeks: PLLA: 70% PLLA + DBP: 90%	PLLA: large numbers of inflammatory cells (12 weeks) PLLA + DBP: Minimal inflammatory reactions (12 weeks)
Schneider *et al.* [9]	rabbits	6 mm calvarial non-critical size, wound was closed with sutures	4 weeks	Radiography Micro-CT histology evaluation with an image software	PLGA/TCP: 34.9 ± 17% Bio Oss: 30.8 ± 14.3% Empty: 28.4 ± 14.9% PLGA: 25.1 ± 14.6%	No sign of inflammation
Schofer *et al.* [30]	rats	5 mm calvarial critical size defects, the wound was closed by suturing the overlaying tissue and skin	4, 8, and 12 weeks	CCT histology evaluation with an image software	4 weeks: PLLA/BMP-2: 31% BS: 4% PLLA: 3% Empty: 1% 8 weeks: PLLA/BMP-2: 48% BS: 6% LLA: 5% Empty: 3% 12 weeks: PLLA/BMP-2: 48% BS: 26% PLLA: 2% Empty: 9%	No sign of inflammation
Shim *et al.* [31]	rabbits	8mm calvarial defects Wound was closed with sutures	2 and 4 weeks	histology	-	2 weeks: cells (mostly connective tissue and inflammatory cells) penetrated the three-dimensional scaffolds. 4 weeks: new bone formation was observed
Yanagida *et al.* [32]	rats	3 mm calvarial defects Wound was closed with sutures	4 weeks	histology	-	PLLA: rarely new bone HAP-mixed/coated PLLA: new bone was more elongated

BG: bioactive glass; BMP-2: bone morphogenetic protein 2; BS: bovine spongiosa; DA: dopamine; CCT: cranial computed tomography; DBP: demineralized bone powder; G: gelatin; HA: hydroxyapatite; MSCT: multislice spiral-computed tomography; SEM: scanning electron microscopy; SIM: simvastatin; TCP, tricalcium phosphate.

#### 3.4.3. New Bone Formation at Different Time Points

##### (1) PLGA

Three studies examined PLGA scaffolds in the calvarial size models in rats [33,36] and rabbits [9]. One out of these three was performed in a critical size defect model [33].

After four weeks, two studies [9,36] reported very different new bone formation. Jiang *et al.*, found new bone formation accounting for 1% when PLGA-HA was used [36], whereas Schneider *et al.*, found new bone formation for 25% (PLGA) and 34% (PLGA/TCP) [9]. The results of the control groups of these two investigations varied as well. The empty defects were filled with 1% [36] and 28% [9] newly formed bone, respectively.

**Figure 2:**
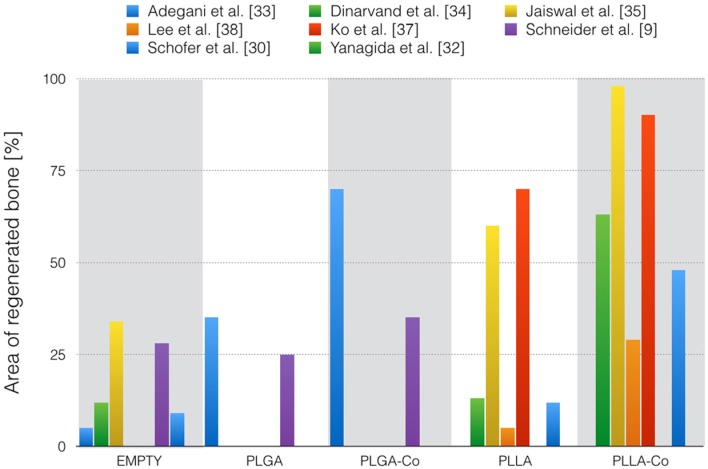
Overview of the different studies with the longest observation period and the following examined groups: empty, mere polymers (PLGA or PLLA), and composites (Co). If more than one composite, only the best result is illustrated.

Examinations after eight weeks were performed in two studies in the rat calvarial model [33,36]. The control groups of these two investigations were approximately in the same range, accounting for 2% [36] and 5% [33] newly formed bone. Pure PLGA scaffolds reported 35% new bone [33]. PLGA scaffolds combined with willmite gained up to 70% [33] new bone formation, whereas only 10% new bone could be formed with HA-SIM [36]. Figure 2 illustrates the area of regenerated new bone for the different studies.

##### (2) PLLA

Examinations investigating PLLA scaffolds were mostly made in the rat calvarial model [30,32,34,35,37]. One study was performed in mice [38] and another one in rabbits [31]. All of these studies evaluating new bone formation worked with a critical size defect model [30,34,35,37,38]. Unfortunately, two studies did not quantify the newly formed bone within these areas in their studies [31,32].

Only one study evaluated the bone formation after four weeks [30]. PLLA scaffolds produced 3% newly formed bone while the empty control group was filled with 1% newly formed bone. In adjunction with BMP-2, PLLA scaffolds showed significantly more new bone formation, accounting for 31%.

One study evaluated bone formation after six weeks [35]. The results showed that mere PLLA scaffolds produced 26% new bone. Blending with gelatin (G), PLLA scaffolds resulted in less bone (13%). Surprisingly, the empty control group showed 30% newly formed bone, thus more bone was formed than in the two groups mentioned before. Significantly more bone formation was found with HA (64%) and in combinations of PLLA with the two substances, namely G and HA (94%).

Four studies [30,34,37,38] evaluated new bone formation after eight weeks and found results with pure PLLA scaffolds reaching from “minimal new formed bone” [37] to 13% newly formed bone [34]. The empty control group defects showed new bone formation between 3% [30] and 12% [34]. Various organic adjunctions combined with PLLA scaffolds augmented bone formation from “bone extent greater than PLLA alone” (PLLA and demineralized bone powder (DBP)) [37] via moderate better results with the following dimensions: PLLA and DBP [37] was slightly better than PLLA alone. The effect was moderate because the authors quantified bone augmentation just in words and not in percentage. PLLA-BG noted 20%, PLLA-HA 23%, PLLA-TCP 44%, and PLLA-HA-BG 63% greater bone amount than the control groups [34]. PLLA scaffolds with BMP-2 showed a new bone fraction area of 48% [30], which is in the range as the TCP adjunction.

After 10 weeks, only one study [35] investigated bone formation with results ranging from 34% in the empty control group up to almost complete defect healing in the PLLA-G-HA (98%). The pure PLLA control showed a defect closure of 60%.

Two studies reported 12-week results [30,37]. Although these two studies showed comparable results at the eight-week control, they showed very different results at the 12-week control. Ko *et al.*, reported about 70% new bone formation with PLLA alone [37], whereas Schofer *et al.*, reported only 12% new bone formation when using pure PLLA [30]. Combination of PLLA with DBP showed almost complete defect closure (90%) [37], while PLLA/BMP-2 reported 48% [30] and a defect closure that did not change between the eighth and 12th weeks.

#### 3.4.4. Biocompatibility Based on the Descriptive Histological Evaluation

##### (1) PLGA

Adegani *et al.*, and Schneider *et al.*, did not find any sign of inflammation in the histological evaluation after four and eight weeks [9,33]. Jiang *et al.*, did not assess biological reaction against the scaffold material [36]. Willmite [33] and TCP [9] showed no other healing performance concerning inflammation reactions in comparison to pure PLGA scaffolds or control groups.

##### (2) PLLA

Four studies found no sign of inflammation [30,34,35,38], whereas Ko *et al.*, reported minimal inflammatory reactions in their histological evaluation within the time from eight to 12 weeks [37]. Shim *et al.*, reported after two weeks about inflammatory and connective tissue cells penetrating PLLA scaffolds [31]. However, they did not report any inflammatory reactions after four weeks. These histological findings were in agreement with biochemical and hematological parameters, indicating no infection due to scaffold implantation [35]. One study did not mention tissue reactions against PLLA scaffolds [32]. However, it remains unclear if any reaction against PLLA was visible or if they simply did not assess this aspect. Regarding the different adjunctions as TCP, HA, BG, DA, BMP-2, and G [30,34,35,38], no distinction was noted between the healing performance of the defects.

## 4. Discussion

The aim of this systematic review was to explore the efficiency of electrospun PLLA or PLGA scaffolds used as bone substitute materials in *in vivo* animal investigations. We focused on the amount of newly formed bone and the biological reactions that these bone substitute materials are provoking *in vivo*.

The 10 studies included in this review differed in terms of material composition, animal model, defect size, time of evaluation, evaluation methods, applied statistical methods, and primary outcome. Due to the pronounced heterogeneity among these studies, precise conclusions could not be made.

All the studies in this review chose calvarial defects for their experiments. This model is a non-load-bearing model (no mechanical stimuli) with rather poor blood supply (limited nutrition) and limited bone marrow (smaller number of progenitor cells) [43]. Consequently, the mere effect of an applied biomaterial can be investigated in a comprehensive manner.

Concerning PLGA scaffolds, conflicting results were found after four weeks. Jiang *et al.*, found remarkably less bone formation in their study within 5 mm defects than Schneider *et al.*, did in 6 mm critical defects. Although PLGA ratios were 85:15 in both studies, the materials differed in fiber diameter and scaffold architecture. While Jiang *et al.*, used scaffolds with smooth nanofibers [36], Schneider *et al.*, used scaffolds in which even the fibers showed a porous structure [9]. TCP nanoparticles increased fiber roughness additionally. Scaffold architecture, such as porosity and pore size, plays a critical role in cell migration and bone formation into a scaffold [44,45]. A high porosity of nanofibers helps cell accommodation and facilitates the efficient exchange of nutrient substances between the scaffold and the environment [46,47,48]. It was examined that a 100 μm pore diameter is necessary for *in vitro* cell migration and a 300 μm pore diameter is necessary for tissue ingrowth and nutrient diffusion [46,49]. However, the effects of scaffold architecture on bone formation can differ depending on the studied materials [50,51]. Furthermore, there is also evidence that scaffold porosity can have no significant effect on bone formation [52]. So, it remains necessary to test each biodegradable scaffold to delineate its influence on bone regeneration. Another reason for the different results could be due to the different animal models for the bone regeneration process. Dog, sheep, goat, pig, and rabbit are the most commonly used models for bone regeneration, whereas dog, sheep, and pig are the models with the highest similarity to humans. Given the considerable dissimilarities with human bone, mice and rats are not counted as desirable models for bone studies [53], though in this review, most of the experiments were done using the rat model.

Concerning PLLA scaffolds, four studies presented eight-week results [30,34,37,38]. The regenerated area of new bone was only slightly more for mere PLLA samples than for the empty control groups. Combinations of PLLA scaffolds with inorganic or organic components showed higher new bone values in comparison to empty control groups or pure PLLA scaffolds in all these studies. Differences between the results are difficult to trace back, though the studies varied in scaffold architectures, defect sizes, and methods of evaluations.

The results of the 12-week controls were conflicting regarding pure PLLA scaffolds [30,37]. To comment on this outcome is demanding because at the eight-week control, the results of the pure PLLA scaffolds of these two studies were in the same range. Differences in study design should have shown an effect in the eight-week control already. One interpretation is that the circumstances in the study continuance changed between eight and 12 weeks in some way in one of the studies.

Focusing on the results from Schofer *et al.* [30], they showed an augmentation of newly formed bone between the four- and eight-week controls. At the 12-week control, the new-formed bone reached larger dimensions in the PLLA and the empty control group but not in the PLLA/BMP-2 group. An explanation of this observation could be that most carriers loaded with BMP-2 show an early burst of BMP-2 release with a reduction of retained BMP-2 release afterwards [54]. However, the BMP-2 release of the incorporated BMP-2 in electrospun PLLA scaffolds seems to be prolonged and with good effects on bone formation within the first eight weeks. The augmentation of newly formed bone ceased afterwards, probably as a consequence of the beginning degradation of the PLLA scaffolds and the adjusted disposability of BMP-2. Similar findings were made by Fu *et al.* and Kim *et al.* [55,56], finding a benefit from incorporating BMP-2 into scaffolds *in vitro* and *in vivo*, namely in the first eight weeks after implantation. Unfortunately, Fu and Kim did not perform a 12-week control, so we do not know if the augmentation of bone volume reached its maximum after eight weeks, similar to the Schofer study.

In contrast to Schofer *et al.*, Ko *et al.*, showed a significant augmentation in new bone formation from the eight-week to the 12-week control. They demonstrated cell ingrowth for up to 12 weeks after implantation. They observed greater calcium content at earlier time points with PLLA/DBP scaffolds *in vitro* compared to the pure PLLA scaffolds. As mineralization is one of the key processes for bone regeneration, the authors concluded an up-regulation of the mineralization process by incorporation of DBP into scaffolds and showed the *in vivo* performance of the combined PLLA/DBP scaffold as mentioned before.

Generally, to improve the biological functionality of synthetic polymers, composite scaffolds have been developed using inorganic substances like HA, TCP, and BG. Bone is composed of nano-assembled collagen type 1 and inorganic HA crystals. Therefore, composite scaffolds with bioactive inorganic particles improve *in vivo* cell interaction. This effect was demonstrated in many *in vitro* and *in vivo* studies [57,58,59]. In this review, compositions of PLGA and PLLA scaffolds with inorganic substances support these findings, showing higher new bone formation than for mere polymeric scaffolds alone [9,33,34,35,36]. Additionally, the use of basic substances, such as HA, can hinder the degradation by neutralizing the buffer media and can even retain a higher percentage of the flexural strength [60]. As mentioned before, methods to evaluate newly formed bone varied in many aspects. Most of the studies used different radiographic methods to quantify newly formed bone areas [9,30,33,34,35,36,37,38]. Furthermore, two of them quantified bone augmentation by two independent radiologists [33,34], while the other authors [9,30,35,36,37,38] used software to analyze new bone volume. These factors should also be considered when comparing the results of the various studies and can partially explain the different results.

Another approach to engineer an effective bone graft material is to integrate substances into the scaffold that are capable of triggering osteogenesis, such as growth factors [61]. In this review, BMP-2 [30], DA [38], DBP [37], and SIM [36] were incorporated into PLLA/PLGA scaffolds. These factors can enhance bone growth compared to pure PLLA/PLGA scaffolds alone and compared to control groups in all studies included in this review, and this supports results from studies made with the named factors *in vitro* and *in vivo* [62,63,64,65,66,67].

PLLA is widely used in medical fields. A disadvantage of PLLA is low cell adhesion on its hydrophobic surface [68]. Another disadvantage is that degradation of PLLA leads to acidic products and these are supposed to produce inflammatory tissue reactions [20,69,70]. However, four out of six studies that assessed tissue reactions histologically did not find any signs of inflammation [30,34,35,38] for PLLA. The same held true for two PLGA studies [9,33]. Two studies reported inflammatory reactions in pure PLLA scaffolds [31,37]. One of these investigations also examined PLLA with DBP [37]. In this combination they assessed only minimal inflammatory reactions in comparison to pure PLLA scaffolds. This might be due to the starting biomineralization process, which could buffer a possible acidic degradation.

## 5. Conclusions

Scaffold composition and architecture determines its biological behavior and degradation characteristics. Therefore, each scaffold has to be tested on its properties *in vitro* and *in vivo*. Nevertheless, general statements can be made:

PLLA and PLGA provide more new bone formation than empty control groups *in vivo*. PLLA/PLGA scaffold compositions with inorganic substances, such as HA, TCP, and BG and/or organic substances such as BMP-2, DA, DBP, and SIM enhance new bone formation additionally. Consequently, a combined scaffold should be favored.

PLLA/PLGA composite scaffolds, especially when combined with basic substances like HA, seem to not induce inflammatory tissue reactions *in vivo*.
